# Case of Labor

**Published:** 1856-12

**Authors:** D. P. Johnson


### REPORT OF A CASE OF LABOR.

BY D. P. JOHNSON, M. D.

On the morning of the 27th of October, I was called to attend Mrs. R. in her first accouchment. She had had, as I learned, considerable uterine pains for the last four or five hours, and slight ones for the last twenty-four hours. I found the os tincæ dilated to about the size of an English shilling, and that the waters (which had been very scant) had been discharged for several hours. Labor progressed rather slowly, but about as rapidly as I could expect, it being her first child, and she on the shady side of thirty. About noon the pains grew more rapid and stronger, and at 2 o'clock, P. M. she was delivered of a male child of 4 ℔s. Finding there was still another child, she was directed to be kept quiet until labor pains should again recur. In about one hour and a half labor pains again set in quite briskly, complicated with Uterine hemorrhage. When the uterus contracted, large quantities of blood were expelled, and when it would relax external hemorrhage would cease, but it was evident that during the intervals between the pains, the cavity occupied by the first child would partially fill up with blood, to be expelled at the next pain. Ergot, frictions and other means were resorted to, to arouse all the expulsive energies of the womb, but the head (presentation being natural) being high up in the superior strait, it advanced but slowly. At every recurrence of pain, the hemorrhage still continued, and the patient becoming very much exhausted, it become evident that something must be done or the patient would be lost. The question occurred as to the propriety of introducing the hand, turning and delivering, or of quieting uterine action. The latter course was taken, and a large dose of morphine administered, and both pains and hemorrhage ceased for nearly twenty-four hours. Labor again set in, and in about a couple of hours she was delivered of a dead child of 8 lbs, double the size of the first. During two or three of the first pains after labor had commenced, the second time, clotted blood came away, but it seemed evident that the mouths of the bleeding vessels had become so constringed and shut up during the long interval of pain, that no serious hemorrhage would probably result. In about thirty minutes after the birth of the last child, a double placenta was expelled, which, from the dark and clotted uterine surfaces, I should suppose, had been patially, if not wholly, detached at the birth of the first child. The patient was very faint for several days, but both mother and child are now doing well.

This case has at least one thing about it peculiar, and that was the second child being just double the size of the first. Was the case properly managed? Had the child been turned and delivered, might it not have been saved, for its movements were distinctly felt by the mother after the birth of the first child? Would turning have been safe in the exhausted condition of the mother, or did I err in not attempting to turn before her strength was exhausted? These and many other questions occurred to me during the management of this case, and should any young reader of the Medical Journal be led to enquire and be better prepared to manage such a case, should he meet it, the object of writing this article will be attained.

